# Ideal Homes

**Published:** 1920-02-07

**Authors:** 


### Ideal Homes.

### Nursery Designs by Queens.

Attention should be drawn to the exceedingly interesting ideal home exhibition organised by the *Daily Mail* and opened at Olympia yesterday. The main floor is covered with buildings in various stages of completeness. They illustrate every variety of modern house construction. Several of them are the direct outcome of experience in rapid construction gained during the war, when the scarcity and high cost of building material drove builders and architects to depart from accepted formulæ.

From the first the organisers of the exhibition have received the assistance of the Ministry of Health, which has erected a real village of houses, each carried up to a level at which the eye can see the plan of the rooms, doors, windows, and passages, and the materials of which the house is built, with a panorama background giving a very pleasant view of the complete village. House-seekers will be able to discuss the arrangement of the rooms and plan of the disposition of their furniture.

The Ministry of Agriculture has also recognised the great public importance of the exhibition, and is installing a full-sized allotment, which will be filled with vegetables growing as they should in June. The crops will include all the home-grown vegetables for the English dinner-table, and will show what can be done in a space of 75 feet by 20 feet.

Electricity in the home comes into its own at this exhibition. The ideal of a home, heated, lighted, cleaned, and kept comfortable by the aid of the electric current, which cooks and " washes up " and in a dozen different ways acts as handmaiden to the modern wife, is thus realised.

The exhibition gives a fair amount of space to the nursery. The Queens of England, Spain, Holland, Belgium, Sweden, and Norway have designed ideal nurseries, and in several instances sent special commissioners from their own households to see that the designs are carried out with due regard to the features special to the country of origin.

While the woman in search of utility will find the exhibition a centre of attraction during the three weeks, February 4 to February 25, during which it will remain open, the theorist and idealist will also visit it for his own special purpose. There are to be conferences arranged by the Royal Institute of British Architects and the International Garden Cities and Town-planning Association.

